# Successful Initiation of Dialysis with a 20-Year-Old Buried Peritoneal Dialysis Catheter: Case Report and Literature Review

**DOI:** 10.1155/2019/5678026

**Published:** 2019-02-18

**Authors:** Ankur Gupta, Susan Lavoie, Brian Blew, Mohan Biyani, Brendan B. McCormick

**Affiliations:** ^1^Division of Nephrology, Department of Medicine, University of Ottawa and The Ottawa Hospital, Canada; ^2^Kidney Research Centre, University of Ottawa, Canada; ^3^Department of Surgery, University of Ottawa and the Ottawa Hospital, Canada

## Abstract

Buried peritoneal dialysis (PD) catheters are typically inserted several weeks or months before the anticipated need for dialysis. Occasionally, renal function unexpectedly stabilizes after the surgery, and a patient may go years before the catheter is needed. We report a case of successful initiation of PD with a twenty-year-old buried catheter. We outline the steps needed to optimize the catheter function and review the benefits of the buried PD catheter.

## 1. Introduction

Since the late 1990s, our centre has inserted buried peritoneal dialysis (PD) catheters using a modified Moncrief-Popovitch technique [1, 2]. The technique was originally described in 1993 as a method for reducing the rate of PD peritonitis [3, 4]. It was postulated that allowing the catheter to heal in the sterile subcutaneous tissue would prevent the primary formation of bacterial biofilm. Delayed exteriorization of the catheter would allow for complete healing of the subcutaneous cuff which would then act as a physical barrier to biofilm formation. Studies did indeed show less biofilm formation with buried PD catheters [5], but this did not consistently translate into reduced rates of PD peritonitis [6–8]. Subsequently, buried PD catheters have been promoted as a way to increase PD utilization by allowing for insertion of a maintenance free access months before it is actually required, similar to early insertion of an arteriovenous fistula [9].

We have reported an ideal time window of exteriorization ranging from 6-weeks to 5-months as this seems to be associated with the lowest risk of primary failure and lowest need for intervention after exteriorization [10]. Furthermore, time to first peritonitis increases with increased length of time embedded suggesting that a well-healed and mature tunnel may lead to less early peritonitis [10]. However, it is postulated that the risk of fibrin plugging, omental wrapping, and catheter migration increases with time the catheter is not in use [10, 11].

## 2. Case

**Figure 1:**
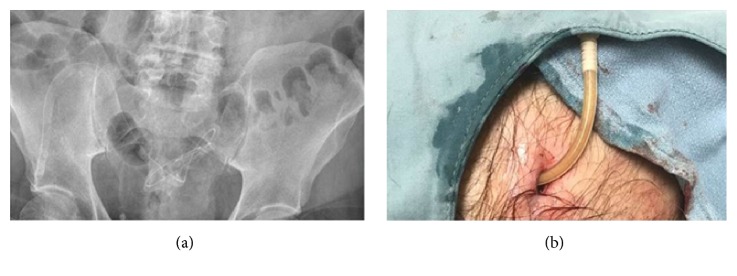
(a) Plain radiograph of the abdomen showing position of catheter prior to exteriorization. (b) Catheter immediately after exteriorization.

A now 76-year-old male underwent buried PD catheter insertion in 1998 at the Ottawa Hospital. He had membranous glomerulopathy and stage 5 chronic kidney disease (CKD) with an estimated glomerular filtration rate (eGFR) of 13 mL/min/1.73m2. Over the ensuing years, he continued to have subnephrotic proteinuria and was managed with perindopril. The renal function remained relatively stable and only began to decline in late 2017. He consented to initiation of PD in February 2018 after his eGFR dropped to 7 mL/min/1.73m2. A plain radiograph of the abdomen showed the PD catheter optimally positioned in the true pelvis (Figure 1(a)).

The exteriorization procedure was performed in the Home Dialysis Unit. A 0.5 cm skin incision was made 2 cm distal to the superficial cuff and a loop of the catheter was mobilized and the fibrin was cleared off the catheter. The distal catheter did not glide out easily. With the assistance of a surgeon, a second incision was made over the distal end of the catheter and it was separated from the subcutaneous tissue by dissection and the end of the catheter was cut off (Figure 1(b)). A large fibrin plug was removed from the lumen of the catheter with push and pull syringe aspiration. The flow remained very sluggish. Tissue plasminogen activator (tPA) was instilled into the catheter and by the following day the inflow significantly improved but outflow was still slow.

Two days later, a cathetergram and guide wire manipulation of the catheter was then arranged through interventional radiology. The initial contrast injection showed the PD catheter localized within a pocket of fibrous tissue communicating with the greater peritoneal cavity along the right pelvic wall. Two angled glide-wires were utilized to clear fibrin out of the lumen of the catheter and a torque cable was then used to flip the draining loop out of the fibrous pocket into the greater peritoneal cavity. Following this, outflow improved and the patient was able to successfully initiate continuous ambulatory PD (CAPD). We have documented a filling time of 6 minutes and drain time of 9 minutes. His clinical course has otherwise been uneventful.

## 3. Discussion

This case highlights a successful exteriorization and initiation of CAPD with a buried catheter that had lain dormant for almost 20 years. To our knowledge, this is the longest reported period of time between implantation of a buried PD catheter and successful initiation of PD. The exteriorization procedure was more challenging than usual as the free end of the catheter required surgical dissection whereas it normally glides out easily. We encountered the expected challenges with fibrin plugging of the catheter, but with a combination of syringe aspiration, tPA instillation, and guidewire manipulation, we were able to successfully initiation PD.

The early placement of a buried PD catheter has numerous advantages [2]. During the time that the catheter is buried, no maintenance is required and the exteriorization is an elective outpatient procedure. The completely healed catheter tunnel at the time of exteriorization allows for the use of full dwell volumes with lower leak risk. Other benefits include flexibility in PD initiation, reduced need for bridging hemodialysis in urgent starts, low rates of infectious complications, and cuff extrusion. While there are occasional reports of visceral perforation with a dormant PD catheter [12], similar reports of trauma have occurred with other methods of catheter insertion [13].

Our current report confirms that there is no upper limit of time after which a buried PD catheter should not be used due to presumed futility. Although we continue to recommend two to six months as the ideal time period for implantation of buried PD catheter prior to use, we strongly suggest that exteriorization of a long buried PD catheter should always be attempted before resorting to other dialysis accesses or modalities.
